# Genetic Diversity, Repeat Motifs, and Natural Selection at the C-Terminal Knob-Associated Histidine Rich Protein (KAHRP) of *Plasmodium falciparum* Clinical Samples from Saudi Arabia

**DOI:** 10.1155/2022/3740889

**Published:** 2022-03-28

**Authors:** Saad M. Bin Dajem, Md Atique Ahmed, Marie Fe F Bohol, Syeda Wasfeea Wazid, Mohammed I. Shafeai, Fuad H. Rudiny, Ali M. Motaen, Kareem Morsy, Hani Alothaid, Ahmed A. Al-Qahtani

**Affiliations:** ^1^Department of Biology, College of Science, King Khalid University, Abha, Saudi Arabia; ^2^ICMR-Regional Medical Research Center, Dibrugarh, Assam, India; ^3^Department of Infection and Immunity, Research Centre, King Faisal Specialist Hospital and Research Centre, Riyadh, Saudi Arabia; ^4^Arogyo Society of Health, Welfare and Support (ASHWAS), Dibrugarh, Assam, India; ^5^Sabya General Hospital, Sabya, Saudi Arabia; ^6^Department of Basic Sciences, Faculty of Applied Medical Sciences, Al-Baha University, Al-Baha, Saudi Arabia; ^7^Department of Microbiology and Immunology, College of Medicine, Alfaisal University, Riyadh, Saudi Arabia

## Abstract

**Background:**

Malaria is still a public health problem in Saudi Arabia specifically in the Jazan region. *Plasmodium falciparum* knob-associated histidine-rich proteins (PfKAHRPs) play an important role in cerebral malaria pathophysiology as well as pathogenesis of *P. falciparum* infections. The repeat region of PfKAHRP C-terminal interaction domain has been found to bind to the infected red blood cells and the vascular endothelium. Thus, this study aimed to assess the allelic variations, genetic diversity, and natural selection acting at the C-terminal PfKAHRP between parasite isolates from Saudi Arabia.

**Materials and Methods:**

The PfKHARP C-terminal interaction domain was successfully PCR-amplified and sequence data from 441 clinical isolates from Saudi Arabia were obtained. The DnaSP v5.10 software was used to determine the genetic diversity, polymorphism, haplotype, and natural selection. Haplotype network analysis was constructed by using the median-joining method in the NETWORK version 5.0.0.1 software.

**Results:**

Alignment and analysis of 441 C-terminal PfKAHRP-deduced amino acid sequences identified 5 genotypes (I–V) based on the decapeptide repeat arrangements (TKEASTSKEA, TKEASTSKGA, TKEASTTEGA, and TKEASTSKRA). Among the repeat types, Type I (49.43%, 218/441) was the most abundant in Saudi Arabia, followed by Type II (48.29%, 213/441). Overall, the nucleotide diversity in the PfKHARP C-terminal region was found to be low in Saudi Arabia (*π* = 0.00142); however, natural selection tests indicated positive selection (dN-dS = 1.64, *P* < 0.05) which was due to the variations within the repeat motifs. Genealogical relationship haplotype network of *PfKAHRP* from 4 different countries (i.e., Saudi Arabia, Iran, Burundi, and India) revealed 1 major shared haplotype cluster (H_1) with samples representative from all 4 countries (Saudi Arabia; *n* = 441, Burundi; *n* = 4, Iran; *n* = 13, and India; *n* = 1).

**Conclusion:**

Since this is the first study to report on genetic diversity of C-terminal PfKAHRP interaction domain and the repeat motifs from clinical samples in Saudi Arabia, it will contribute towards the rational design of antiadhesion drug therapies for *P. falciparum* malaria.

## 1. Introduction

Saudi Arabia has made significant progress in reducing the annual number of malaria cases thereby inching closer towards malaria elimination; however, it still remains a public health problem in the Jazan region [1]. A recent cross-sectional study (with samples from April 2018 to January 2019) from the Jazan region reported 94.3% of the malaria infections were due to *Plasmodium falciparum* [1]. Though locally transmitted malaria cases decreased between 2000 and 2014, a spike of 5,382 cases was reported with 272 confirmed local transmission [2, 3]. *Anopheles arabiensis* is the dominant vector for malaria in the region which usually peaks following the rainy season [4]; however, other competent species reported in the region include *An. dthali, An. stephensi, An. superpictus, An. culicifacies*, and *An. fluviatilis* [5–7]. Cerebral malaria is one of the major causes of mortality in *P. falciparum*-infected patients. The pathophysiology involves the cytoadherence of parasite knob-associated histidine-rich proteins (KAHRPs) and infected red blood cells (RBCs) within the brain cells of infected patients. These knob-associated proteins within *P. falciparum* parasites remodel the host cell membrane to assure ionic homeostasis, thereby increasing nutrient uptake for their own survival [8, 9]. Knob proteins form protrusions on the infected erythrocyte surface through which they adhere to uninfected erythrocytes and the microvascular endothelium [9]. This parasite-induced cytoadherence phenomenon is the hallmark of malaria severity in patients where-in a high number of accumulated infected erythrocytes within the microvasculature disrupts blood flow, causes inflammation, and oxygen depletion leading to organ damage [10].

One more key parasite protein family known as erythrocyte membrane protein-1 (EMP1) members play a vital role during cytoadherence in association with KAHRP in *P. falciparum*. Structural analysis of PfKAHRP and PfEMP-1 indicated formation of complexes with host erythrocyte membranes for formation of knobs and facilitating cytoadherence [8]. Targeted gene knock-out studies on the C-terminal PfKAHRP showed that repeat region is essential for knob formation within parasitized erythrocytes [11]. The PfKAHRP protein, therefore, plays an important role in cerebral malaria pathophysiology as well as pathogenesis of *P. falciparum* infections.

Structurally, the PfKAHRP protein constitutes 3 domains, the N-terminal histidine-rich domain, the central domain, and the C-terminal repeat domain, which has decapeptide repeats [6] and the latter two domains are found to be immunogenic [7]. Although *P. falciparum* knob structures are unique, all *Plasmodium* species that infect primates have KAHRP-like proteins [12]. The genetic diversity of PfKAHRP has not been studied in clinical samples from Saudi Arabia. Sequence comparison from various worldwide samples showed polymorphism exists mostly within the C-terminal domain and a few variant forms of KAHRP have been observed [8]. Thus, the primary objective of this study was to understand the genetic variants of the PfKAHRP C-terminal domain from 441 clinical samples from Saudi Arabia, characterise the repeat motifs, and compare with available worldwide samples (Iran, India, and Burundi). This is the first study to report on the diversity of the PfKAHRP C-terminal domain from Saudi Arabia. The findings of the study will contribute to the knowledge of KAHRP variants from Saudi Arabia and will be helpful towards rational design of antiadhesion therapies for *P. falciparum* malaria.

## 2. Materials and Methods

### 2.1. Study Site

Finger-prick blood samples on filter paper were collected from RDT-positive *P. falciparum* patients attending health centres in Jazan province. Informed consent was obtained from all patients before blood sampling. Thick and thin blood smears were also obtained from the patients for microscopic detection using Giemsa stain. Detailed information on parasite counts, age, and sex of 441 patients enrolled in the study are given in Supplementary Table 1. This study was approved by the Ethical Review Committee of Research in Ethical Review Board (ERB) of King Fahad Central Hospital (KFCH), Jazan.

### 2.2. DNA Extraction and PCR Amplification

DNA extraction was carried out from filter papers using QIAamp DNA blood mini kit according to manufacturer's instructions. The C-terminal domain of PfKAHRP gene (PF3D7_0202000, from 1888 nt to 2290 nt) was amplified using nested PCR (KAHRPFOR-1 and KAHRPREV-1 for Nest 1 PCR and KAHRPFOR-2 and KAHRPREV-2 for Nest 2 PCR amplification). To facilitate the PCR product sequencing, the inner primers were linked to M13 forward and reverse primers (Table 1). The following PCR amplification conditions were used: for round one, 1.5 µl DNA template, 1X final concentration of GoTaq Green Master Mix (Promega, Madison, WI, USA), 0.3 µM each primer, in 30 µl final volume. The cycling conditions were 95°C for 5 mins followed by 35 cycles at 95°C for 45 s, 60°C for 30 s, and 72°C for 45 s, then, a final extension at 72°C for 5 min. For nested PCR, 1 µl of round one amplicon was used, and 1X final concentration of GoTaq Green Master Mix, 0.3 µM each primer in 25 µl final volume were used. Cycling parameters were 95°C for 5 mins, followed by 5 cycles of 95°C for 20 s, 62°C for 20 s and 72°C for 45 s, 25 cycles of 95°C for 20 s, 58°C for 20 s and 72°C for 45 s, followed by a final extension at 72°C for 5 min. PCR products were run in 2.5% agarose gel to confirm their size. PCR products were directly sent for DNA sequencing using M13 forward and reverse primers and only consensus sequences were used for analysis.

### 2.3. Sequence Data

All raw sequence generated in this study were initially analysed and trimmed using the SeqMan software (DNA Star) and best quality sequences were converted to fasta format for analysis. Published *PfKAHRP* sequence data originating from Iran [13], Burundi [14], and India [15] were also included for comparative analysis. The sequences generated from this study were submitted to NCBI with the following accession number OK070812-OK071252.

### 2.4. Genetic Diversity and Natural Selection

Sequence diversity (*π*), number of polymorphic sites, number of synonymous and nonsynonymous substitutions, haplotype diversity (Hd), and number of haplotypes (H) within the *PfKAHR*P sequences was determined by using the DnaSP v5.10 software [16]. Natural selection was determined at the intra- and interpopulation levels. At the intrapopulation level, natural selection was determined by calculating the rates of synonymous substitutions per synonymous site (dS) and nonsynonymous substitutions per nonsynonymous site (dN) as computed by using Nei and Gojobori's method and robustness were estimated by the bootstrap method with 1000 pseudo replicates as implemented in the MEGA 5.0 software [17]. Difference between dN and dS were determined by applying codon-based *Z*-test (*P* < 0.05) in MEGA software v5 with 1000 bootstrap replications [17]. The Tajima's *D*, Fu and Li's D^*∗*^, and F^*∗*^ neutrality tests were performed as implemented in the DnaSP v5.10 software.

### 2.5. Genealogical Network Analysis

Genealogical relationships between the *PfKAHRP* nucleotide haplotypes were constructed using the median-joining method in NETWORK software (version 4.6.1.2, Fluxus Technology Ltd, Suffolk, UK). Sequences obtained in this study were submitted to NCBI.

## 3. Results

### 3.1. Deca-Peptide Repeat Types from Saudi Arabia

**Figure 1:**
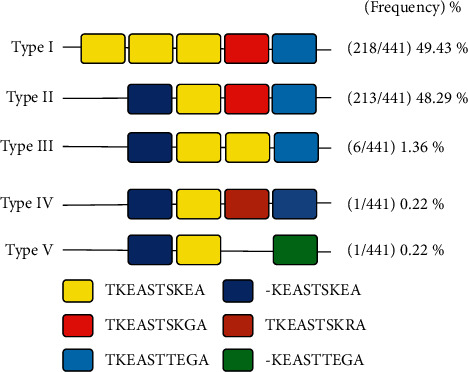
Repeat types (I–V) observed within C-terminal PfKHARP clinical isolates from Saudi Arabia and their frequency. Each deca and nonapeptide motifs observed within 441 sequences is color coded.

Based on deduced amino acid alignments of 441 PfKAHRP clinical samples from Saudi Arabia, we observed five different genotypes depending on decapeptide repeat motif (TKEASTSKEA)n and its arrangement with 3 decapeptide motifs TKEASTSKGA, TKEASTTEGA, and TKEASTSKRA and two nonapeptide motif KEASTSKEA and KEASTTEGA (Figure 1). Among the 5 genotypes, we found the Type I (49.43%, 218/441) to be the most abundant among the clinical samples from Saudi Arabia, followed by Type II (48.29%, 213/441), Type III (1.36%, 6/441), Type IV (1.36%, 6/441), and Type V (1.36%, 6/441) (Figure 1).

### 3.2. Polymorphism, Nucleotide, and Haplotype Diversity within *PfKAHRP* Gene Sequences

Alignment and comparison of 487 nucleotide sequences (from 4 countries: Saudi Arabia, Iran, India, and Burundi) of the C-terminal region of *PfKAHRP* gene identified 6 polymorphic sites (yielding 5 haplotypes) out of which, 5 sites were nonsynonymous. There were 4 parsimony informative sites and 2 singleton variable sites. The overall nucleotide diversity was marginally higher (*π* = 0.00158 ± SD 0.00029) when compared to sequences from Saudi Arabia (*π* = 0.00142 ± 0.00004). The haplotype diversity of *PfKAHRP* was higher (Hd = 0.477 ± SD 0.013) compared to overall combined diversity of all 4 countries (Hd = 0.106 ± SD 0.019).

### 3.3. Natural Selection in *PfKAHRP*

To determine whether natural selection contributes to the polymorphism in the *PfKAHRP* gene, the average difference of (dN-dS) was evaluated. We found positive values (dN-dS = 1.64) at the C-terminal domain (Table 2) indicating dN > dS for Saudi Arabian samples. We confirmed this by using codon-based *Z*-test for positive selection using Nei and Gojobori's method and found significant positive selection (dN > dS, *P* < 0.05) (Table 2). We also used this test statistics on all samples from 4 countries and found similar results (dN > dS, *P* < 0.03). However, Tajima's *D*, Li and Fu's F^*∗*^, and D^*∗*^ statistics were found to be negative for *pftrap* C-terminal domain (Table 2).

### 3.4. Genealogical Haplotype Network Analysis

**Figure 2:**
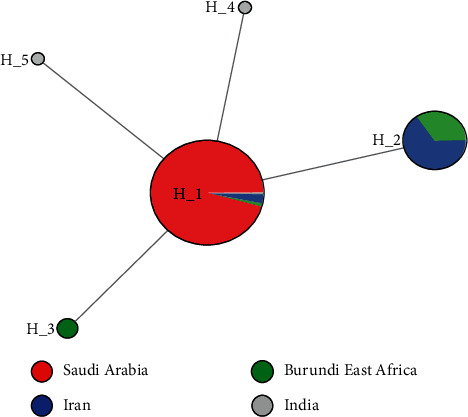
*PfKAHRP* haplotype network generated from 4 different countries. The size of the circles representing each haplotype is proportional to the number of sequences that correspond to this haplotype. Distances between nodes are arbitrary.

A haplotype network tree was constructed to understand the phylogeography and genealogical relationships with *PfKAHRP* haplotypes from 4 different countries (i.e., Saudi Arabia, Iran, Burundi, and India). The network tree revealed 2 shared haplotypes (H_1 and H_2) (Figure 2) among these haplotypes, H_1 formed the major haplotype cluster with samples representative from all 4 countries (Saudi Arabia; *n* = 441, Burundi; *n* = 4, Iran; *n* = 13, and India; *n* = 1). Haplotype 2 (H_2) was shared between Burundi and Iran. Haplotype (H_3) was a unique cluster with samples originating only from Burundi, East Africa. Two unique haplotypes (H_4 and H_5) were observed with samples from India.

## 4. Discussion

Peptide repeats are very common in malarial antigens and thus are the major source of antigenic variations which helps the parasite to evade host immunity [18–21]. Parasite proteins with tandem repeats are excellent targets for host immune response too. One of the most complex antigen with repeats which is also highly immunogenic is the circumsporozoite protein (CSP) and it has been found to be immunogenic in multiple species [19–22]. Both N- and C-terminal region of PfCSP are relatively conserved in worldwide samples and natural selection are observed within the C-terminal Th2R and Th3R epitope binding domains [23]. Recently, the World Health Organisation (WHO) has recommended the use of RTS, S/AS01 (RTS, S), a CSP-based world's first malaria vaccine for children in sub-Saharan African countries and in regions where there is moderate to high malaria transmission [24]. Considering the importance of characterising antigenic targets with repeats, the current study was designed to characterise the KAHRP repeat motifs, determine genetic diversity, and natural selection from Saudi Arabia and compared with three countries, i.e., Iran, India, and Burundi available from the NCBI database. The study revealed that there were five genotypes (Type I–Type V) within clinical isolates of Saudi Arabia based on the arrangement of the decapeptide tandem repeat motif (TKEASTSKEA)n. Type I (218/441, 49.43%) and Type II (213/441, 48.29%) were the major genotypes observed within Saudi Arabia. It would be interesting to know whether these genotypes have varying degree of binding to host erythrocytes and endothelium leading to severe disease in some patients. Antibody response to these tandem repeat types needs to be explored too. Higher nucleotide diversity was observed within the repeat region with evidence of positive natural selection indicating possible parasite-host interaction to evade host immunity. Similar higher levels of nucleotide diversity with strong positive selection within the repeat regions were also observed in *P. ovale* CSP [25]. These studies indicate towards functional constraints towards at the host-parasite interaction domains. Most antigens which are expressed at the merozoite apex, for e.g., *P. falciparum* and *P. knowlesi* merozoite surface proteins (MSPs): MSP4, MSP7, MSP1, Pk41, and MSP1P show varying levels of polymorphisms and strong natural selection due to exposure to host immune response [26–32]. Among these merozoite antigens, MSP1 and MSP2 contain repeat motifs which are immunogenic at the same time are associated with the merozoite surface [18]. PfMSP1 antibodies which recognise the repeat regions also show parasite inhibitory activities in vitro [33]. Haplotype network analysis showed that Saudi Arabian samples formed a major cluster along with samples from other countries indicating low levels of polymorphism within the interaction domain. The nucleotide diversity of *PfKAHRP* from Saudi Arabia as well as all 4 countries together were of similar levels indicating that it is the SNPs associated with the variations in the repeat region was responsible for diversity. These findings of the present study will contribute to the knowledge of KAHRP variants from Saudi Arabia and contribute towards the rational design of antiadhesion therapies for *P. falciparum* malaria.

## 5. Conclusion

This is the first study to report on the *PfKAHRP* genotypes from Saudi Arabia and shared major haplotypes circulating within the region. Since *PfKAHRP* is a key participant in severe malaria pathology and include repeat motifs that are recognised by the immune system, understanding these sequences is critical for understanding severe disease, treatment, and prevention. More such studies are necessary to understand the major parasite variants within and to understand the functional aspects for designing antiadhesion therapies against *P. falciparum*.

## Figures and Tables

**Table 1:** Primers used for amplification and sequencing of *PfKHARP* gene.

Primer name	Primer sequence
KAHRPFOR-1	GGA AAT GAC GGT GAA GGA GA
KAHRPREV-1	CAG AGA TTA TTT AAC CAC AGC ATC C
KAHRPFOR-2	***GTA AAA CGA CGG CCA GT*** G AAA CAA AAA ACA CCG CTG
KAHRPREV-2	***CAG GAA ACA GCT ATG ACC*** GTA CTG CAT TAG CTC CTG TAG TTG

M13 sequences are in bold and italics.

**Table 2:** Estimates of nucleotide diversity, natural selection, haplotype diversity, and neutrality indices of C-terminal *PfKAHRP*.

Origin	No. of samples	SNPs	No. of haplotype	Diversity±SD		dN-dS	Codon-based *z* test	Taj D	Fu and Li's D^*∗*^	Fu and Li's F^*∗*^
				Haplotype	Nucleotide					
Saudi Arabia	441	4	5	0.477 ± 0.013	0.00142 ± 0.00004	1.64	*P* < 0.05	−0.3	−1.9	−1.64
All 4 countries	487	6	5	0.106 ± 0.019	0.00158 ± 0.00029	1.83	*P* < 0.03	−1.23	−1.2	−1.4

SNPs: single nucleotide polymorphisms; SD: standard deviation.

## Data Availability

The data generated from this study have been submitted to NCBI with the accession numbers OK070812–OK071252.

## Abbreviations

KAHRP: Knob-associated histidine-rich proteins.

## Supplementary Materials

Supplementary Materials Supplementary Table1: baseline characteristics of 441 *P. falciparum* infected patients enrolled in the study.
