# Supplementary material for: Genetic Diversity, Repeat Motifs, and Natural Selection at the C-Terminal Knob-Associated Histidine Rich Protein (KAHRP) of Plasmodium falciparum Clinical Samples from Saudi Arabia
Source: J Trop Med. 2022 Mar 28;2022:3740889. doi: 10.1155/2022/3740889 (PMC8979686; doi:10.1155/2022/3740889)
Supplement: Supplementary Materials — Supplementary Table1: baseline characteristics of 441 P. falciparum infected patients enrolled in the study. [file 3740889.f1.docx]

Supplementary Table1: Baseline characteristics of 441 *P.* *falciparum* infected patients enrolled in the study.

| Variables | Value |
| --- | --- |
| *Age* |  |
| Mean ± SD | 28.01 ± 13.95 |
| Median (25th-75th) | 25.50 (19.0-36.75) |
| *Sex* |  |
| Male count % | 375 (85.03%) |
| Female count % | 66 (14.97%) |
| *Parasite density** |  |
| Low | 68 (15.42%) |
| Moderate | 94 (21.32%) |
| High | 279 (63.26%) |

***** WHO. Basic Malaria Microscopy. 2. Geneva: World Health Organization; 2010.
